# Complete mitochondrial genome of *Caranx equula* (Perciformes, Carangidae): genome characterization and phylogenetic analysis

**DOI:** 10.1080/23802359.2018.1491333

**Published:** 2018-07-13

**Authors:** Yuena Sun, Tianjun Xu

**Affiliations:** a Key Laboratory of Exploration and Utilization of Aquatic Genetic Resources (Shanghai Ocean University), Ministry of Education, Shanghai, China;; b International Research Center for Marine Biosciences at Shanghai Ocean University, Ministry of Science and Technology, Shanghai, China;; c Key Laboratory of Freshwater Aquatic Genetic Resources, Ministry of Agriculture, Shanghai, China

**Keywords:** Carangidae, *Caranx equula*, mitochondrial genome, phylogenetic analysis

## Abstract

*Caranx equula* is an important marine fish which due to its commercial values. However, some wild populations of *C. equula* are in danger because of the overfishing and environmental pollution. In this study, the complete mitochondrial genome of *C. equula* was firstly determined. The complete mitochondrial genome is 16,607 base pairs in length, and contained 13 protein-coding genes and 2 ribosomal RNA genes, 22 tRNA genes and 2 main non-coding regions. Overall A + T content was 52.9%. In addition, a phylogenetic tree was constructed using the complete mitochondrial genome and showed that *C. equula* clustered in a clade and formed a sister relationship with *Caranx ignobilis* belonged to the family of Carangidae.

*Caranx equula* belongs to the family Carangidae, which is widely distributed in tropical waters of Indo-Pacific. It is a reef-associated fish and famous for its edibleness and economic value. Although it has these significant values, many of its wild populations are in danger due to the ecological damages, overfishing, and marine pollution. Now the conservation of *C. equula* need to be pay an important attention. In this study, the complete nucleotide sequences for the mitochondrial genome of the *C. equula* was present. And to examine the evolutionary position of *C. equula,* a phylogenetic tree using Bayesian inference analysis was constructed. The present result will facilitate further investigations on the taxonomic resolution and genetic conservation of Carangidae.

The specimen of *C. equula* was collected from the waters around the East China Sea (29.9'N, 122.2'E) and was kept at the Museum of Marine Biology at Zhejiang Ocean University. Eight primer pairs were designed to amplify the complete mitochondrial genome of *C. equula* (Supplemental Table S1). The sequence fragments were assembled. The tRNA genes were identified by their proposed clover-leaf secondary structure and anti-codons. All the sequences collected from GenBank (Supplemental Table S2) were used to construct phylogenetic trees by Bayesian inference analysis.

The mitochondrial DNA of *C. equula* is a closed double-stranded circular molecule of 16,607 bp (GenBank accession number: KX373635) and contained 13 protein genes, 2 rRNA genes, 22 tRNA genes, 1 putative control region (CR), and 1 light strand replication origin (O_L_; Supplemental Table S3). The base composition of the complete mitochondrial genome was A 27.9%, C 30.4%, G 16.7%, and T 25%, with a slight A + T bias of 52.9% (Supplemental Table S4), which was similar to other fishes mitochondrial base content (Liu et al. 2013; Cheng et al. 2012). The 13 protein-coding genes had a strong bias against G (8.4%) at the third codon position, which was typical in vertebrate mitochondrial genomes (Ishiguro et al. 2001). Most of these genes are encoded on the H-strand, except for the ND6 gene and eight tRNA genes (ND6, tRNA^Gln^, tRNA^Ala^, tRNA^Asn^, tRNA^Cys^, tRNA^Tyr^, tRNA^Ser^, tRNA^Glu^, and tRNA^Pro^), which are encoded on the L-strand.

The 12S and 16S rRNA genes of *C. equula* which are similar to other vertebrates, located between tRNA^Phe^ and tRNA^Leu^ (UUR) genes, and are separated by the tRNA^Phy^. All the protein-coding genes started with ATG, except for COI used GTG, ATP6 used ATA as the start codon. The stop codon of seven protein-coding genes (ND1, COI, ATP8, ATP6, ND4L, ND5, and ND6) is TAA. The remaining four genes seemed to end in two incomplete stop codons, TA- (COIII) and T- (ND2, COII,ND3, ND4, and Cytb), which were completed via post-transcriptional polyadenylation (Ojala et al. 1981).

The 22 tRNA genes which have two forms, including tRNA^Ser (UCN and AGY)^ and tRNA^Leu(UUR and CUN)^, scatter throughout the genome and range from 57 to 74 bp in size and the gene arrangement is typically as in most vertebrates. Three tRNA clusters (IQM, HSL, and WANCY) were well conserved in *C. equula* as in other vertebrate mitochondrial genomes. Fourteen tRNA genes were encoded by H-strand, while the remaining eight tRNA genes (tRNA^Gln^, tRNA^Ala^, tRNA^Asn^, tRNA^Cys^, tRNA^Tyr^, tRNA^Ser^, tRNA^Glu^, and tRNA^Pro^) were encoded by L-strand. The total length of overlaps and intergenic spacers were 18 and 82 bp, ranging from 1 to 7 bp and from 1 to 36 bp per location, respectively. As in most vertebrates, two non-coding regions can be found in *C. equula* mitogenome, an O_L_ within the WANCY region including five tRNA genes (tRNA^Trp^, tRNA^Ala^, tRNA^Asn^, tRNA^Cys^, and tRNA^Tyr^), which can fold into a stem loop secondary structure with the conserved motif 5′-GCCGG-3′. The control region in *C. equula* mitogenome is determined between tRNA^Pro^ and tRNA^Phe^ and this characterization is consistent with those of other teleost (Jin et al. 2013; Wei et al. 2013; Xu et al. 2011).

To investigate the phylogenetic position of *C. equula* among closely related fishes, a phylogenetic tree was constructed with other teleost mitochondrial genome sequences using Bayesian inference analysis. The mitochondrial genome sequence of *Megalops atlanticus* was used as outgroup. Phylogenetic analysis result demonstrated that *C. equula* clustered in a clade and formed a sister relationship with other species belong to the family of Carangidae (Figure 1). This complete mitochondrial genome can be used for population genomic studies and the data will provide fundamental information for the genetic conservation and the taxonomic resolution of Carangidae.

**Figure 1.**
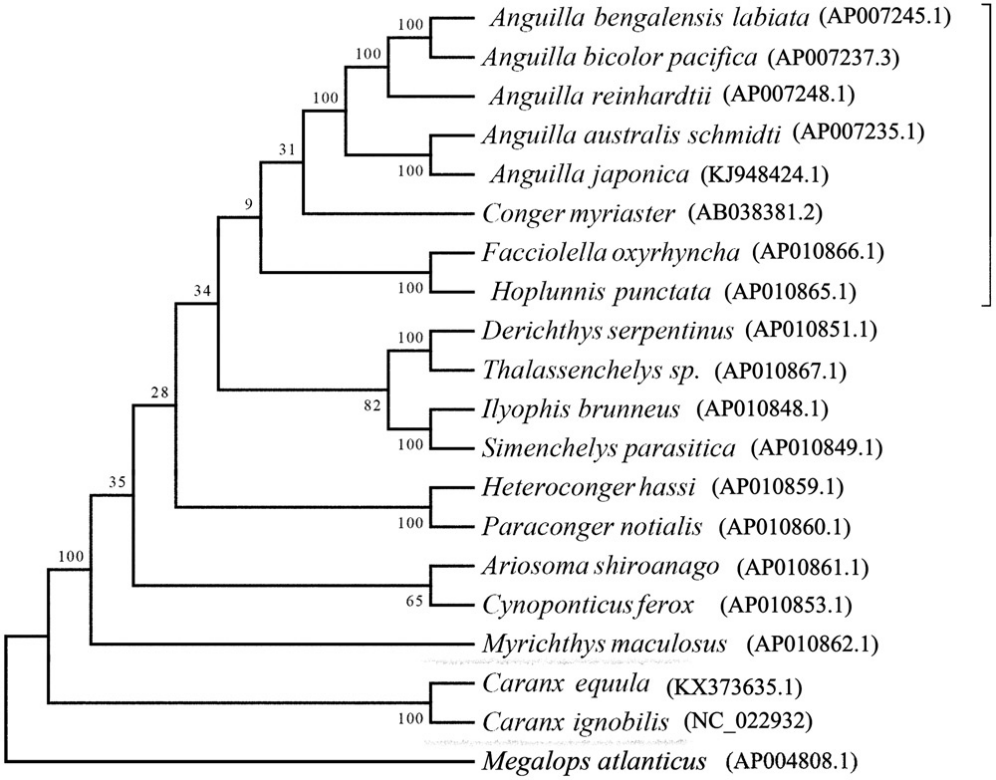
Phylogenetic tree based on the complete mitochondrial genome sequences was constructed by using Bayesian method. The numbers in topologies represent Bayesian posterior probability values.
